# Retargeting Strategies for Oncolytic Herpes Simplex Viruses

**DOI:** 10.3390/v8030063

**Published:** 2016-02-26

**Authors:** Gabriella Campadelli-Fiume, Biljana Petrovic, Valerio Leoni, Tatiana Gianni, Elisa Avitabile, Costanza Casiraghi, Valentina Gatta

**Affiliations:** Department of Experimental, Diagnostic and Specialty Medicine, University of Bologna, Bologna 40126, Italy; gabriella.campadelli@unibo.it (G.C.-F.); bily.p@hotmail.it (B.P.); valerio.leoni2@unibo.it (V.L.); tatiana.gianni3@unibo.it (T.G.); elisa.avitabile@unibo.it (E.A.)

**Keywords:** oncolytic HSV, retargeting, HER-2

## Abstract

Most of the oncolytic herpes simplex viruses (HSVs) exhibit a high safety profile achieved through attenuation. They carry defects in virulence proteins that antagonize host cell response to the virus, including innate response, apoptosis, authophagy, and depend on tumor cell proliferation. They grow robustly in cancer cells, provided that these are deficient in host cell responses, which is often the case. To overcome the attenuation limits, a strategy is to render the virus highly cancer-specific, e.g., by retargeting their tropism to cancer-specific receptors, and detargeting from natural receptors. The target we selected is HER-2, overexpressed in breast, ovarian and other cancers. Entry of wt-HSV requires the essential glycoproteins gD, gH/gL and gB. Here, we reviewed that oncolytic HSV retargeting was achieved through modifications in gD: the addition of a single-chain antibody (scFv) to HER-2 coupled with appropriate deletions to remove part of the natural receptors’ binding sites. Recently, we showed that also gH/gL can be a retargeting tool. The insertion of an scFv to HER-2 at the gH N-terminus, coupled with deletions in gD, led to a recombinant capable to use HER-2 as the sole receptor. The retargeted oncolytic HSVs can be administered systemically by means of carrier cells-forcedly-infected mesenchymal stem cells. Altogether, the retargeted oncolytic HSVs are highly cancer-specific and their replication is not dependent on intrinsic defects of the tumor cells. They might be further modified to express immunomodulatory molecules.

## 1. The Need for Non-Attenuated Cancer-Specific Oncolytic HSVs

More than twenty years have passed since a herpes simplex virus (HSV) was assayed for the first time in the experimental therapy of human glioblastomas, with the aim of killing the tumor cells and reducing the tumor burden [1]. This initiated the oncolytic virotherapy era. Subsequent efforts were aimed at improving the safety profile, by reducing the intrinsic virulence of the virus. In essence, safety was obtained at the expense of virulence through single or multiple deletions [2]. Most frequently, the oncolytic HSVs are deleted in the neurovirulence gene γ_1_34.5, and gain cancer specificity from the fact that a number of cancer cells are defective in mounting the innate protein kinase R (PKR) response to the virus [3,4]. Additional deletions were included to further improve the safety profile [5]. Attenuated natural mutants, exemplified by HF10, were also selected [6]. These viruses have been and still are being tested in clinical trials [7,8,9,10,11,12,13,14,15,16,17,18], for a review [19,20,21]. Viruses were safely administered intratumorally at doses up to 3 × 10^9^ plaque forming units (PFU) [12]. In general, they exhibit a high safety profile and exert antitumor activity against a variety of tumor types [21]. However, because of their attenuation, robust growth occurs only in tumors with defects in innate response, autophagy, apoptosis, *etc.*, or with high tumor proliferation rate.

The need for less restricted oncolytic HSVs led to the design of the first immunotherapeutic oncolytic HSVs. Rabkin and coworkers [22] engineered a replication-competent attenuated HSV expressing interleukin 12 (IL-12), designed to induce local and systemic antitumor immunity and favor a Th1 response. Markert, Gillespie and Whitley engineered HSV recombinants deleted in the γ_1_34.5 gene and expressing IL-12, IL-4 or IL-10 [23,24]. A phase 1 trial to test the efficacy of the IL-12-encoding virus in glioblastoma patients is now ongoing (ClinicalTrials.gov identifier NCT02062827). Coffin and coworkers engineered the gene encoding granulocyte macrophage-colony stimulating factor (GM-CSF) in a ∆γ_1_34.5 virus (OncoVEX^GM−CSF^), to enhance dendritic cell recruitment and boost the host immune response against the tumor [25]. In a phase III clinical trial, the patients with metastatic melanoma lesions who received repeated intratumoral inoculations of the virus, later renamed talimogene laherparepvec (T-VEC), showed a statistically significant increased durable response rate and an increased overall survival (slightly statistically not significant), when compared to the patients that received subcutaneous injections of GM-CSF alone [26]. The U.S. Food and Drug Administration approved this product for the treatment of melanoma lesions in the skin and lymph nodes [27], and the European Medicine Agency recommended it.

## 2. Overview of Tropism Retargeting Based on Modification of gD

An alternative strategy to more potent oncolytic HSVs, capable of replicating and killing cancer cells independently of intrinsic defects in the tumor and overcoming most of the limitations imposed by the tumor heterogeneity, is to render the virus highly cancer-specific by retargeting its tropism to cancer-specific receptors of choice and detargeting from natural receptors. Such viruses carry no deletion and preserve the full-blown lytic capacity of wt-HSV. In the HSV field, this idea was first introduced by Glorioso and co-workers at a time when the number and the specific roles of the HSV glycoproteins involved in virus entry was still unclear [28].

**Figure 1:**
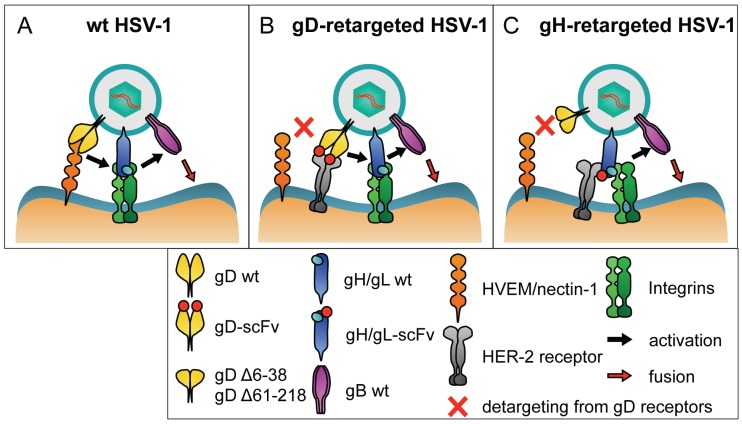
Schematic drawings showing the principle of tropism retargeting of oncolytic HSVs to cancer-specific receptors. (**A**) Essential interactions in entry of wt-HSV. gD is activated by the interaction with one of its natural receptors, nectin1 or HVEM. The activation is transmitted to gH/gL, which are also activated by an integrin (αvβ6 or αvβ8). The activation is finally signaled to gB, the fusogenic glycoprotein that carries out the fusion of the virion envelope with the cell membrane, either the plasma or an endosomal membrane; (**B**) Retargeting through genetic modifications in gD. gD carries the deletion of AA regions 6–38, or 61–218, which prevents the interaction of gD with its natural receptors. The deleted sequences are replaced with scFv to HER-2, for retargeting to HER-2. The interaction of the scFv-gD chimera with HER-2 activates gD. Activation is then transmitted to gH/gL and to gB; (**C**) Retargeting through genetic modifications in gH. gD carries the deletion of AA 6–38 to prevent the interaction with its natural receptors. gH carries an scFv to HER-2 inserted at its N-terminus. The interaction of the chimeric gH with HER-2 and with integrins is sufficient to activate gH/gL, in the absence of an activation signal from receptor-bound gD. gH/gL activation is signaled to gB.

It is now known that HSV entry is a multistep process that involves an attachment glycoprotein gC, plus four essential glycoproteins gD, gH/gL and gB (Figure 1A). gD is species-specific and is thought to be the major determinant of HSV tropism. gH/gL and gB represent the conserved fusion apparatus across the *Herpesviridae* family. gB has structural features typical of viral fusion glycoproteins [29,30,31,32,33,34]. Entry initiates with gD binding to one of its receptors: nectin-1, HVEM and modified heparan sulphates [35,36,37,38]. This binding triggers conformational changes in gD, including the dislodgement of the C-terminus ectodomain, exposing the profusion domain [39,40,41,42,43]. The speculative part of the model envisions that activation spreads through intermolecular signalling from the receptor-activated gD to gH/gL and then to gB, or through the recruitment of the glycoproteins to a macromolecular complex [44]. gH/gL undergo conformational changes with displacement of gL [44]. gB executes the fusion between virion and cell membranes. It is unclear whether gH/gL are simply intermediates in the activation cascade or participate in the fusion event [45,46].

A breakthrough in HSV retargeting strategies was the identification of gD as the virion glycoprotein to be modified [47,48]. Zhou and Roizman engineered IL-13 in gD N-terminus. IL-13 is the ligand of the IL-13 Receptor 2α, expressed in glioblastoma. The resulting virus was effectively retargeted to the IL-13R 2α and detargeted from HVEM [48]. Retargeting to urokinase plasminogen activator receptor (uPAR) was also explored [49]. A split form of gD or a mutation in S34 in gD were introduced for the purpose of detargeting the viral tropism from nectin-1 [47,49,50]. However, the effect of the S34 substitution was not universal [50] , and the split gD virus grew at relatively low titers.

**Figure 2:**
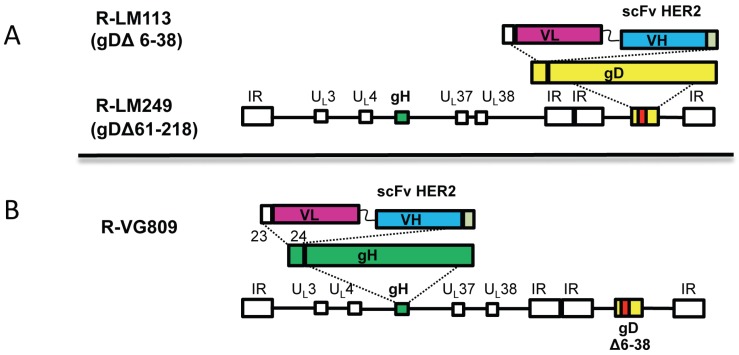
Schematic drawings of the genome backbones of oncolytic HSVs retargeted through genetic engineering of gD or of gH. (**A**) Backbone of two gD retargeted HSVs, R-LM113 and R-LM249. Sequence arrangement of HSV-1 genome shows the inverted repeat sequences as rectangular boxes. The scFv-HER2 sequence (VL-linker-VH) is inserted in place of AA 3–38, or 61–218 of gD; (**B**) Backbone of a gH-retargeted HSVs. Sequence arrangement of HSV-1 genome shows the inverted repeat sequences as rectangular boxes. The scFv-HER2 sequence (VL-linker-VH) is inserted between AA 23 and 24 of gH. In addition, all recombinants carry LOX-P-bracketed p-Belo-BAC inserted between UL3–UL4, and the sequences for fluorescent marker, inserted either within the BAC sequences, or between UL37–UL38 regions.

In our laboratory, we choose HER-2 (human epidermal growth factor receptor 2) as the target cancer-specific receptor. HER-2 is a member of the EGFR (epidermal growth factor receptor) family, overexpressed in breast and ovarian cancers, gastric carcinomas, glioblastomas, *etc.* [51]. Two fully retargeted oncolytic HSVs were generated (Figure 1 and Figure 2). The gD regions that were deleted for detargeting purposes differ between these two oncolytic viruses (Figure 1). R-LM113 gD is deleted of the AA 6–38 N-terminal portion [50,52]. R-LM249 gD is deleted in the core region (AA 61–218) [53]. In both viruses, the single chain antibody (scFv) to HER2 replaced the deleted portions of gD (Figure 2). The scFv to HER2 was derived from trastuzumab, a humanized MAb now in clinical practice, and interacts with HER-2 at a high affinity (29.3 nM) [54]. Overall, the *in vitro* growth of the two retargeted HSVs in cancer cells is about one log lower than that of the wt-virus [50,53]. A direct comparison with replication and killing ability of ∆γ_1_34.5 viruses has not been performed yet. The safety profile of HER-2-retargeted oncolytic HSVs is predicted to be high. Thus, the deletions in gD rule out any possibility that the virus can revert to the wt-virus. In addition, in the unforeseen case that a same cell is double infected with a retargeted virus and a wt-virus, recombination events can only lead to the two parental viruses.

In preclinical experiments, we demonstrated a therapeutic effect of R-LM113 and R-LM249 against human breast and ovarian cancers, and against a murine model of HER2^+^ glioblastoma [50,52,53,55,56]. R-LM249 exerted a therapeutic effect against peritoneal and brain metastases of ovarian and breast cancers after intra-peritoneal injections [55]. R-LM249 can be administered systemically by means of carrier cells, and exerts therapeutic effects against lung and brain metastases [57]. The full retargeting observed in cell cultures holds true also in animals. We found evidence of viral replication exclusively in neoplastic deposits even in mice in which R-LM249 was administered through the i.p. route [55]. Even though the mouse system did not faithfully recapitulate the situation in humans (only the tumor cells expressed human HER-2), R-LM249 failed to infect off-target HER-2-negative cells.

Glorioso and Grandi chose EGFR as the target receptor expressed in a number of cancers, including glioblastoma. For detargeting purposes, gD carried the deletion of AA 2–24 (detargeting from HVEM) and one AA was mutated (Y38C, detargeting from nectin-1). The scFv to EGFR replaced the deleted portion of gD [58]. Interestingly, the gD modifications were combined with mutations (D285N e A549T) in gB, which increase the rate of entry. The EGFR-retargeted virus exerts therapeutic effects in an orthotopic mouse model of primary human glioma [58]. Taking into account the Glorioso-Grandi and our own approaches, it would seem that a general detargeting-retargeting strategy is the deletion and replacement of the AA 6–38 region in gD.

Parenthetically, as noted by Miest and Cattaneo [19], even though HSV entry is more complex than that of the measles virus (MV), the tropism of both viruses can be modified using similar strategies. The basic idea that emerged is that retargeting is readily achieved with enveloped viruses, likely because the structure of glycoproteins imposes less constraints than the rigid structure of the capsid proteins. The best results were obtained with the enveloped viruses that encode two distinct glycoproteins for attachment and for fusion, exemplified by MV and HSV [59]. A positive feature of retargeted oncolytic HSVs and oncolytic MVs is that they can be detargeted from the natural receptors [60,61].

## 3. Tropism Retargeting Based on Modifications of gH

Recently, we challenged the idea that any retargeting strategy for HSV would entail modifications to gD and asked whether gH can also serve as a retargeting tool. We were prompted by our recent discovery that HSV gH/gL interacts with two interchangeable receptors, αvβ6- and αvβ8-integrin. These receptors participate in the HSV entry process by promoting virus endocytosis, and the displacement of gL from the gH/gL heterodimer; most likely, the latter is part of the process of gH activation. We reasoned that if gH/gL serve as receptor-binding glycoproteins, then a hetelogous ligand engineered in gH might redirect the viral tropism. It was known that gH can tolerate an insert at the N-terminus. Specifically, Krupp and Mettenleiter isolated a viable gL-minus pseudorabies virus recombinant carrying a gD-gH fusion [62]. Cohen and Eisenberg generated a similar HSV recombinant carrying a gD-gH chimera, in which the entire ectodomain of gD was fused at the N-terminus (residue 22) of gH [63]. This chimera was capable of complementing a gD^−/−^ gH^+^ virus or a gD^+^ gH^−/−^ virus. These findings provided evidence that gH tolerates an insert at the N-terminus, and that the gD in the chimera may enable gH activation. However, since gH/gL carry a binding site for gD, the activation from gD located *in cis*, rather than *in trans*, was not totally surprising. However, it did not predict whether the gH/gL activation could be achieved through a heterologous ligand, and whether this would result in modification to the HSV tropism.

We engineered the scFv to HER-2 at the N-terminus of gH, between AA 23 and 24 (Figure 2). AA 1–20 constitute the signal sequence, absent from mature gH. Two recombinants were generated. R-VG803 carries a wt-gD. R-VG809 carries the deletion of AA 6–38 region in gD for detargeting purposes [64]. The redirected tropism of R-VG803 and R-VG809 to HER2 was documented as the ability to infect the receptor-negative J cells transgenically expressing a single receptor, *i.e.*, HER-2, HVEM, or nectin-1, and as the ability to infect human or animal cell lines expressing HER-2. Both R-VG803 and R-VG809 infected J-HER2 cells, and the infection was blocked by trastuzumab—the MAb to HER-2 from which the scFv was derived. Thus, they use HER2 as the sole receptor, *i.e.*, as the portal of entry, in the absence of a gD receptor (Figure 1C). In contrast to R-VG803, R-VG809 failed to infect J-nectin-1, or J-HVEM cells, as well as a panel of HER-2-negative human and animal cells, as expected. This property is consistent with the detargeting effect of the partial deletion in gD. In conclusion, we have defined the site of insertion of scFv to HER2 in gH and ascertained that it can be coupled with gD deletions for detargeting from natural receptors [64]. These findings support two key conclusions: (i) it is possible to modify HSV tropism through the insertion of a heterologous ligand in gH; and (ii) a gH-retargeted-HSV is infectious even in the absence of a gD receptor capable of activating gD.

## 4. Additional Retargeting Strategies

To restrict oncolytic viral replication to tumor cells, transcriptional retargeting was investigated. This approach consists in placing a key viral gene under the control of a promoter that is active in cancer cells only. For example, placing the γ_1_34.5 gene under the control of Musashi1 promoter restricts its expression to malignant glioma cells [65]. A similar approach was pursued for retargeting to prostate tumors: the essential α47 gene was transcriptionally regulated to achieve efficient viral replication in prostate cells [66]. Additional examples include hepatocellular carcinoma specific promoters [67] and regulation of the essential α4 by hypoxia-inducible factor (HIF)-responsive promoter [68]. Finally, micro-RNA target sequences were employed to further decrease off-target infection. Thus, Glorioso and Grandi inserted in the α4 gene the target sequences of miR-124, expressed in neurons and absent from the glioblastoma cells, to protect healthy tissue against virus replication [69].

## 5. Systemic Delivery of Retargeted Oncolytic HSVs by Means of Carrier Cells

The ideal route of delivery for oncolytic viruses in humans carrying metastatic disease is the systemic one. Viruses belonging to different species exhibit intrinsic differences in their ability to spread through the systemic route. Furthermore, given that the majority of systemically administered oncolytic viruses are rapidly cleared by parenchymal organs and inactivated by non-specific and specific defense systems, it may be very difficult, and most likely unsafe, to reach the high blood concentrations required to achieve the locally active concentrations. Further yet, there would be production problems [70]. In the case of oncolytic HSVs, the efficacy of the intravenous (i.v.) systemic route of administration has been attempted in a limited number of preclinical studies [71,72,73,74]. In a phase I clinical trial, the delivery through hepatic artery was safe and showed some efficacy [15,75,76].

A recent approach to circumvent delivery obstacles envisions the use of carrier cells, which package the viral cargo and deliver it to the tumor. Initial studies employed irradiated tumor cells, which have not been pursued because of safety concerns [77,78,79,80]. The mesenchymal stem cells appear as a promising carrier. They accumulate within the tumor stroma because of hypoxic conditions and of tumor-associated expression of inflammatory chemokines. A limit is that the cancer-specificity of the oncolytic viruses may prevent infection of these cells. In addition, the adoptively transferred mesenchymal stem cells might contribute to feed the tumor. So far, mesenchymal stem cells have been assayed as a carrier of oncolytic HSV only in a few studies for intraperitoneal delivery or for local delivery to brain tumors [79,81,82,83,84,85,86,87].

The use of mesenchymal stem cells as carriers of retargeted oncolytic viruses has not been attempted, likely because these cells exhibit scarse-null expression of the targeted receptors. Recently, we reported that mesenchimal stromal cells (MSCs) from different sources can be forcedly infected with an HER2-retargeted oncolytic HSV, by exposing the virion-MSC mixtures to the fusogenic agent PEG 6000 [57]. Progeny viruses spread from MSCs to cancer cells *in vitro* and *in vivo*. We evaluated the organ distribution and therapeutic efficacy in two murine models of metastatic cancers, following a single i.v. injection of infected MSCs. The highest concentration of carrier-cells and of viral genomes was in the lungs, the organs where the viral and cellular genomes accumulated at higher amounts for anatomical reasons. Viral genomes persisted throughout the body for at least two days. The growth of ovarian cancer lung metastases in nude mice was strongly inhibited, and the majority of treated mice appeared metastasis-free. The treatment also significantly inhibited breast cancer metastases to the brain in the NOD scid gamma mice, and reduced by more than one-half the metastatic burden in the brain [57]. Clearly, the therapeutic effects reflected the advantages of the system, *i.e.*, the viral cargo was multiplied within the cells, and was shielded from defensive systems. We did not detect any increase in tumor burden in mice treated with uninfected MSCs, ruling out major side effects of MSC administration in mice.

## 6. Conclusions

The combined oncolytic and immunotherapy approaches have made a remarkable impact on the virotherapy field. It is now possible to treat tumors locally, and, at the same time, induce therapeutic effects at distal sites, by breaking the host tolerance to the tumor. However, there remains a need for less attenuated, highly cancer-specific oncolytic viruses, capable of replicating and spreading despite intrinsic defects and heterogeneity in the cancer cell population [2]. In addition, only a fraction of patients respond to immune checkpoint blockade therapies.

Our approach to non attenuated oncolytic HSVs has been the design of viruses which gain safety from the retargeting of the viral tropism to cancer-specific receptors. Here, we reviewed that, in addition to gD, gH also can serve as a tool for retargeting, and that retargeted oncolytic HSVs can be administered systemically by means of forcedly infected mesenchymal stem cells. Interestingly, the finding that infection with the gH-retargeted oncolytic HSV can take place in the absence of gD-mediated activation impacts the current model of HSV entry based on a cascade of glycoprotein activation. The data argue that the activation that propagates from receptor-bound gD can actually be substituted by the binding of the scFv to HER2 to its cognate receptor, HER-2. In addition, the retargeting studies open the possibility to engineer double retargeted oncolytic HSVs. Patients carrying tumors with low level HER-2 expression are non eligible for therapy with the anti-HER2 antibodies. These tumors often express alternative receptors of the EGFR family, and patients could benefit from a double retargeted oncolytic HSV. Double-retargeted oncolytic HSVs would also prevent the selection of cancer cells negative for the targeted receptor, a phenomenon that occurs frequently in patients treated with targeted therapies, e.g., with human antibodies to the cancer receptors.
